# Regression Analysis and Optimum Values of Austempering Affecting Mechanical Properties of Compacted Graphite Iron

**DOI:** 10.3390/ma17205024

**Published:** 2024-10-14

**Authors:** Aneta Jakubus, Marek Sławomir Soiński, Piotr Mierzwa, Grzegorz Stradomski

**Affiliations:** 1 Faculty of Technology, Jacob of Paradies University, 66-400 Gorzów Wielkopolski, Poland; 2 Faculty of Production Engineering and Materials Technology, Czestochowa University of Technology, 19 Armii Krajowej Av., 42-201 Czestochowa, Poland

**Keywords:** compacted graphite iron, austempering, austenitization, vermicular cast iron, AVGI, RSM, regression models

## Abstract

The study examined the effect of heat treatment parameters of compacted graphite iron (CGI) on the mechanical properties of the material. The microstructure was characterized using optical microscopy, scanning electron microscopy (SEM), and transmission electron microscopy (TEM). Three levels of heat treatment parameters were adopted considering the orthogonal test plan 2^4^. The effects of austenitizing temperature and time and austempering on tensile strength, yield strength, and elongation were analyzed. Polynomial regression was chosen because it extends linear regression and allows for modeling more complex, nonlinear relationships between variables. Total regression models were determined for each dataset. The models for tensile strength (R_m_) had an approximately 82% coefficient of determination, for yield strength (R_0.2_) around 50%, and for elongation (A_5_) around 80%. For optimization, the response surface method (RSM) was used. The results obtained were compared with the proposed mathematical models. The ANOVO results showed that austempering temperature (T_pi_) had the greatest effect on each parameter studied. The optimal conditions for the analyzed parameters, assuming tensile strength and yield strength at the maximum level and an elongation of about 0.7%, are obtained for the following heat treatment parameters: T_γ_ = 890 °C; T_pi_ = 290 °C; τ_γ_ = 120 min; τ_pi_ = 150 min.

## 1. Introduction

As a casting alloy, cast iron is the most widely used material for all kinds of machine and equipment components. In 2021, its total production was about 68 million tons, accounting for 70% of the world’s foundry output [1]. It is not surprising that this material is subjected to various modifications to improve its strength and performance properties, as it is much cheaper to produce compared to steel or cast steel. In recent years, ductile iron (DI) has been rising to prominence. In 2000, global production of this type of cast iron was 13 million tons, and in 2021, it had already reached approximately 25 million tons, representing an increase of about 100% [1]. A number of researchers have investigated DI highlighting the use of this alloy at low temperatures [2], the distribution and influence of individual alloying elements [3,4,5,6,7,8], subjecting it to hot plastic deformation and compression [9], applying coatings to extend the life of the alloy [10], or remelting the surface with a laser beam [11]. The production of DI involves the procedure of spheroidizing and modifying low-sulfur cast iron, as it is well known that as long as sulfur and oxygen are in the molten bath, magnesium (which is used for spheroidizing) causes deoxidation and desulfurization, and spheroidizing occurs only after a certain period of time [6,12,13,14]. In the case of DI, one of the methods used to improve strength properties is to change the matrix to ausferritic. This matrix can be obtained either by controlling the chemical composition [15] or by heat treatment.

The two-stage heat treatment involves austenitizing the cast iron to obtain austenite in the matrix, followed by rapid cooling to a temperature in the range of 250–400 °C and holding for the time necessary for austempering to occur [16]. Austempering and isothermal transformation in cast iron results in a structure consisting of acicular ferrite and residual austenite [17]. A number of researchers have analyzed the effect of heat treatment parameters on graphite precipitation, matrix, strength properties, abrasive wear, or thermal fatigue of austempered ductile iron (ADI) [18,19,20,21]. This type of cast iron has rapidly gained importance because the spheroidal shape of the graphite improves selected strength and performance properties. In [18], the authors analyzed the effect of heat treatment of ADI on hardness. The austenitizing temperature in this case was constant at 950° for 120 min. The austempering temperatures and times were variable at 290 °C, 320 °C, 350 °C, and 380 °C as well as 30, 60, 90, and 120 min. The results indicated that the highest hardness is obtained with an austempering temperature of 290 °C for 30 min. This was due to the thickening of ferrite needles and a higher proportion of residual austenite. The authors of the paper [22] austenitized cast iron with 0.87 Cu and 00.25 Mo at 910 °C for 90 min, then tempered it in a salt bath at temperatures ranging from 350, 370, 390, and 410 °C, obtaining the highest ductility of the material tempered at 410 °C, and the highest tensile strength for cast iron tempered at 350 °C. Jakubus et al. [23] found that as the temperature of austempering increases, there is a tendency for the ratio of ferrite in the matrix to increase, thereby decreasing the ratio of austenite. Given its ductility, increasing the ratio of ferrite in the ausferritic matrix resulted in improved ductility of the cast iron at the expense of its tensile strength. The performance properties of ADI, such as corrosion resistance and abrasive wear, were studied by Krawiec, H. et al. [24]. The researchers analyzed samples austenitized at 900 °C for a period of 2 h and then austempered at 280 °C and 430 °C for 30 and 120 min. It was found that the depth of scoring increased as the austempering time and temperature increased.

With the creation of ductile iron in 1948 came compacted graphite iron, which at first was considered undesirable. The main problem in the CGI production process is controlling the process due to the relatively narrow process window [25]. However, it proved to have good mechanical properties and high resistance to dynamic temperature changes, which placed it between gray cast iron with flake graphite and ductile iron [26,27,28,29]. Compacted graphite iron was not used for mass production of automotive parts until the late 1960s. The increased role of CGI is related to the fact that it can be applied to castings operating under temperature gradients and heat shocks [30,31,32]. A number of researchers have studied it for abrasive wear intended for components such as piston rings, brake discs, cylinder blocks, etc.

A similar heat treatment that ductile iron undergoes to produce ADI is used to boost the properties of CGI. In the study [33], the researchers compared this heat treatment of both ductile iron and compacted graphite iron. The authors austenitized the samples at 900 °C for 120 min, followed by isothermal quenching at 300 °C, 370 °C, and 440 °C, maintaining the time within the range of 5 to 300 min. They found that after isothermal quenching at 300 °C for 150 min, the ausferrite needles were thinner in vermicular iron compared to ductile iron. This is associated with the larger iron–graphite interface areas characteristic of compacted graphite irons.

Austempered vermicular cast iron (AVCI), however, is not as widely produced as ADI; this is probably due to much less knowledge of the effects of the chemical composition and processes occurring in the material during austempering on the structure (matrix, morphology of precipitates) and mechanical and functional properties of the alloy [34]. In order to better understand these processes, a number of researchers are analyzing this issue. For example, in the study [35], the researchers examined the influence of isothermal quenching parameters on the microstructure and mechanical properties of AVGI cast iron. Austenitization was carried out at 900 °C for 60 min, and austempering was carried out at 275, 325, and 375 °C and for time periods of 30, 60, 90, and 120 min. The austempering temperature was found to have a significant effect on the mechanical properties of cast iron; as the austempering temperature decreased, tensile strength and hardness increased while elongation decreased. Other authors [36] carried out heat treatment of CGI at the following parameters: T_γ_ = 900 °C for 90 min and T_pi_ = 325 °C for 120 min. Compared to the “baseline” CGI, heat treatment increased hardness and improved abrasive properties. In [37], AVGI tempered under four different variants was examined and compared with poured cast iron. It was observed, among other things, that the surface roughness of the castings decreased as the temperature of austempering decreased.

The general goal of multiple regression is to quantify the relationships between multiple independent (explanatory) variables and the dependent (outcome) variable. Our objective is to determine whether the heat treatment parameters (four parameters) affect the mechanical properties of the alloy. The aim is to understand how to control these parameters to achieve the desired strength properties. For instance, how does the duration of isothermal quenching affect tensile strength (R_m_), and are there any dependencies between the parameters that influence R_m_? It is well known that the temperature of isothermal quenching influences Rm and yield strength (R_0.2_), and, to achieve low strength and hardness in CGI while increasing elongation, a high temperature is selected because this results in a higher amount of retained austenite. At low temperatures (<350 °C), the alloy achieves high strength. The relationships and correlations between heat treatment parameters provide insight into how to conduct the treatment to achieve the desired mechanical properties.

Regression models are used specifically to calculate approximate values based on existing data. They can predict various outcomes, but the main purpose is for the model to estimate the dependent variable’s value (result) based on the independent variables (factors). These models allow for predicting and analyzing results that are close to real-world values based on the data we already have.

The purpose of this study was to analyze the effect of heat treatment parameters—austenitizing and austempering of compacted graphite iron—on the mechanical properties of the material. In a series of studies, researchers focus on linear analyses that determine the influence of a single heat treatment parameter on selected properties. Therefore, this work incorporates a multi-criteria analysis, which allows for the assessment of correlations between processing parameters and the specified properties. A number of researchers have studied various manufacturing processes in a similar manner. For example, the paper [38] analyzed the hardness of A383 alloy using the cooling slope casting process. The response surface methodology is used to analyze the impact of individual parameters and their interactions on casting hardness. The researchers analyzed the effect of parameters, i.e., slope length, slope angle, and pouring temperature on melt hardness, using ANOVA. The hardness of the alloy was observed to increase with longer slope lengths and higher pouring temperatures, but it decreased as the slope angle increased. A predictive regression model was developed to forecast hardness based on changes in the combination of input process parameters. The optimal hardness was achieved at a pouring temperature of 596 °C, a slope length of 596.5 mm, and a slope angle of 27°. The authors of [39] analyzed in a similar way in order to improve the machining process of Cu/Mo-SiCP composites produced by powder metallurgy. In this study, Cu/Mo-SiCP composite materials with varying reinforcement ratios were produced using powder metallurgy, and their machinability was subsequently analyzed. In the machinability experiments, the selected process parameters included cutting speed (vC), feed rate (f), depth of cut (aP), and reinforcement ratio (RR). Two levels of each parameter were chosen according to Taguchi’s L8 orthogonal array, and response surface methodology (RSM) was applied for parametric optimization. In a similar way, the authors of work [40], using the Plackett–Burman design (PBD) to optimize the adsorption process, assessed the adsorption capacity of four agro-wastes (AWs) derived from pistachio nutshells (PNSs) and aloe vera leaves (AV), as well as multi-walled carbon nanotubes (MWCNTs). Saikaew et al. [41] determined the optimal proportions of cast iron scrap, steel scrap, carbon, and ferro silicon that affect the hardness and quality of cast iron. They conducted the analysis using a mixture of experimental design, analysis of variance, and response surface methodology combined with a desirability function. Fajdek-Bieda A. [42] proposed the optimization of the geraniol transformation process in the presence of natural mineral diatomite as a catalyst. The variables used in the study were temperature, catalyst concentration, and reaction time. The process and response functions were defined as the conversion of geraniol (GA) and the selectivity of its conversion to beta-pinene (BP), respectively. The results enabled the identification of the optimal set of parameters that yielded the highest GA conversion and selectivity for BP production. In a similar way, the authors of paper [43] analyzed the wear and friction of hemp fiber-reinforced polymer for brake discs, and in paper [44], the performance of water jet cutting of tool steel using various abrasives.

## 2. Materials and Methods

### 2.1. Baseline Material

The “baseline” cast iron for subsequent treatment into compacted graphite iron was smelted in a 4000 kg acid-lined medium-frequency crucible induction furnace. After the input materials were loaded into the furnace and melted, about 2000 kg of liquid metal was taken—into an appropriately heated vat—and poured into an acid-lined mains frequency induction furnace with a capacity of 3000 kg. Three mortars from the German company SKW Giesserei GmbH (Unterneukirchen, Germany) were used to produce the cast iron.

When the liquid metal reached a temperature of 1533 °C, and the slag was drawn off, the pouring of cast iron from the furnace into a “slender” ladle with a lid was initiated. Concurrently with the pouring of the metal, VL(Ce)2 mortar was administered to the metal stream in order to “condition” the cast iron, i.e., to pretreat the alloy (this included deoxidizing the cast iron). Prior to the start of metal pouring, DENODUL 5 cast iron vermiculite mortar was placed in a heated slender ladle.

After pouring the liquid baseline cast iron, the “slender” ladle was transported to the modification bench, where the SRF 75 graphitizing modifier was applied to the metal stream after drawing off the slag. This operation was performed during the pouring of liquid metal from the “slender” ladle into the pouring ladle. After the graphitization modification, the pouring ladle filled with cast iron was transported to the pouring station, where the molds made of self-hardening compound were poured. U-shaped sample ingots with test wall thicknesses of 25 mm (according to [45]) were cast. A detailed description of the creation of cast iron can be found in the works [46].

**Figure 1:**
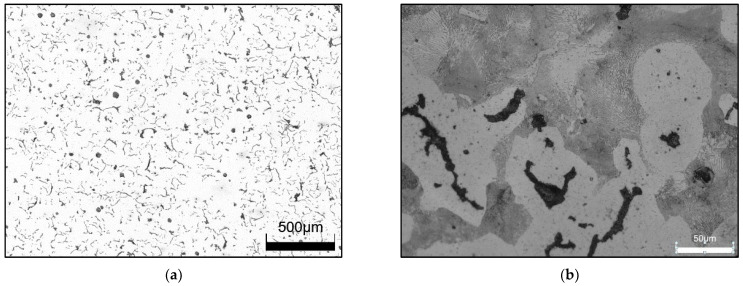
Cast iron with vermicular graphite as-cast state; (**a**) shape and size of graphite particles, nonetched specimen; (**b**) microstructure of cast iron, metallographic specimen etched with Nital.

Table 1 shows the chemical composition of the CGI produced. The content of basic elements was determined using a FOUNDRY–MASTER emission spectrometer from WAS AG. Figure 1 shows the shape and size of the graphite and the microstructure of the CGI. The Olympus DSX1000 digital microscope was used for the observations.

Observations showed that the samples taken from the test portions of the test ingots had a vast majority of vermicular graphite precipitates, and the proportion of spherical graphite was at several percent. The proportion of ferrite in the structure in the samples studied was slightly greater than 50%; the remainder of the matrix was perlite. It should be noted that despite approximately 1% copper content, the proportion of perlite was less than expected, while the literature data show that the addition of 0.82% copper in cast iron is sufficient to obtain a fully perlite matrix [48,49]. Metallographic observations of the cast iron samples taken did not reveal the presence of gas bubbles, non-metallic inclusions, or cracks in them. The results of metallographic tests provided the basis for accepting the produced cast iron as a material that meets the requirements for compacted graphite iron.

### 2.2. Heat Treatment

From the test parts of the IIb test ingots [46], strength samples were taken with the diameter of the measuring part of 25 mm. The samples were subjected to heat treatment by heating them to the appropriate austenitization temperature (i.e., in the range of 850–960 °C [24]), followed by austempering in a salt bath to a specified temperature (i.e., in the range of 250–400 °C [24]) and holding them for a designated time. The purpose of this treatment is to achieve an ausferritic matrix, the formation of which is associated with the nucleation and growth of plates in the austenitic matrix. The samples were austenitized and then austempered in a salt bath to a certain temperature and held there for a predetermined time. A test stand at the “Odlewnie Polskie” in Starachowice was used to carry out heat treatment. It consisted of an Elterma resistance furnace and a quenching tank, and the tempering agent was a salt bath.

CGI heat treatment was carried out based on factorial planning. The statistical treatment of the data produced in the study was treated using Minitab 21.4.3.0 software (Pennsylvania State University, Pennsylvania, PA, USA). A composition master plan was used to determine the effects of heat treatment parameters on tensile strength (R_m_), yield strength (R_0.2_), and elongation (A_5_). With the chosen plan, the number of experiments performed and the level of individual interaction factors (values of output variables) were limited. The interaction terms were mutually independent, i.e., uncorrelated, which enabled maximum information to be obtained about the studied object. A factorial design 2^4^ was used to determine primary effects and quadratic and two-way interaction effects. This plan is used to identify the mathematical model describing the relationship between variables, and the function describing the relationships usually takes the form of second-degree polynomials defined by the following formula:

(1) $$\hat{y}=b_{0}+\sum_{i=1}^{n}b_{i}x_{i}+\sum_{1\leq x\neq j}b_{ij}(x_{i}x_{j})+\sum_{i=1}^{n}b_{ii}x_{i}^{2}$$

where $\hat{y}$ denotes the calculated value of the parameter, *b_o_*—absolute term, *b_i_*—regression coefficients of linear terms of the model, *x*_i_—linear terms of the factors, *b_ij_*—regression coefficients of the two-coefficient terms, *x_i_x_j_*—interaction terms of two factors, *x_i_* and *x_j_*, *b_ii_*—regression coefficients of quadratic terms, and $x_{i}^{2}$—quadratic terms of factors.

**Figure 2:**
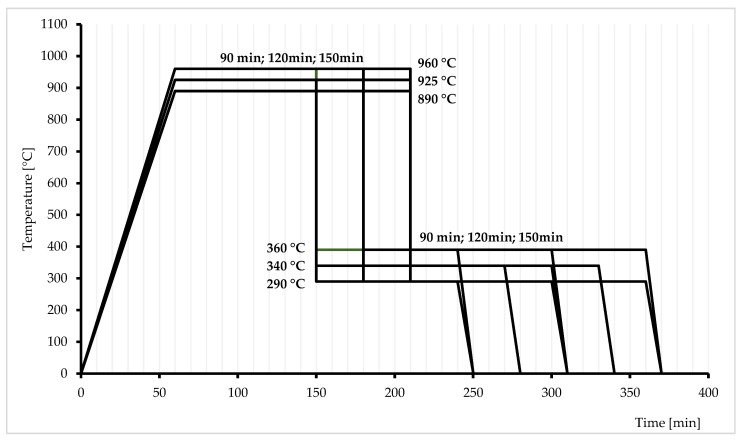
Schematic diagram of heat treatment cycles.

The study was based on four independent variables, such as austenitizing (T_γ_) and austempering (T_pi_) temperatures and austenitizing (τ_γ_) and austempering (τ_pi_) times. Figure 2 shows a diagram of the heat treatment performed. The following designations and ranges were used:

- X_1_—austenitization temperature (T_γ_) from 890 °C to 960 °C;
- X_2_—austempering temperature (T_pi_) from 290 °C to 390 °C;
- X_3_—austenitization time [min] (τ_γ_) from 90 min to 150 min;
- X_4_—austempering time (τ_pi_) from 90 min to 150 min.

The experiment consists of three parts of levels of planned variables. In each part of the system, the values are coded accordingly. The first part is the “kernel of the plan”, in which the factors take two values labeled “−1” and “+1.” There are 16 experiments in this part (see Table 2; Experiments 1 through 16). The second part is the “star points,” in which the studied variables take two levels labeled “α” and “−α” (in our case, 1 and −1), with a total of 8 experiments (Table 2, Experiments 17 through 24). The third part of the system is the “plan center,” where the examined variables take the designation “0.” The selection included 3 experiments performed under the same assumptions (see Table 2, Experiments 25 through 27). A total of 27 experiments were planned.

### 2.3. Strength Properties

**Figure 3:**
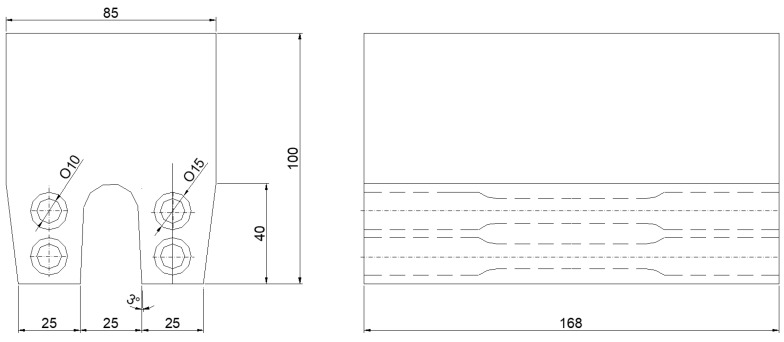
Place of location of strength samples in the test part of the Type IIb test ingot.

The study determined the tensile strength of cast iron both before and after heat treatment. To this end, samples for strength testing were machined from the test portions of cast Type IIb test ingots after they had been cut off from the head. The location of the tensile test samples in the test section of the ingot is shown in Figure 3.

The diameter of the measuring part of the tensile test samples was deliberately increased from the usual 10 mm to 12 mm due to the possibility of scale formation during the annealing process. The excess was then removed after the heat treatment. The samples intended for testing were 160 cm long, and the diameter of the gripping cross-section was 18 cm. Four trials were conducted for each variant of the experiment. The minimum sample size was determined based on statistical methods, assuming an acceptable estimation error of 20 MPa. Mechanical properties were tested using a ZWICK 1488 (ZwickRoell, Ulm, Germany) strength testing machine at the Department of Foundry Engineering, Częstochowa University of Technology.

Based on the results of mechanical properties tests, mathematical models describing R_m_, R_0.2,_ and A_5_ of AVCI as a function of heat treatment parameters were determined. Different regression models can be used to assess four independent variables. The options include linear regression, polynomial regression, and logistic regression. Polynomial regression was chosen because it extends linear regression and allows for modeling more complex, nonlinear relationships between variables. Total regression models were determined for each dataset. To perform the analysis, optimization was carried out using multivariate statistical techniques. The response surface methodology (RSM) was employed, which is based on fitting a polynomial equation to the input (experimental) data. This method is applied when the response of interest is influenced by several variables. Initially, a central composite experimental design was chosen, as it limited the number of required experiments. Regression equations were established in the form of polynomial functions containing quadratic terms, describing the relationships between the selected heat treatment parameters and the mechanical properties of the alloy (tensile strength, yield strength, and elongation). The function was chosen because the study aims to estimate first-order and second-order effects, as well as interactions. A regression is considered significant if at least one of the coefficients *b*_1_, *b*_2_, … *b_n_* of the model is significantly different from zero or if the coefficient of determination (R^2^) is significantly different from zero. The next step is to apply analysis of variance (ANOVA) and determine the optimal conditions, as described in the work [50]. Pareto charts of standardized effects and 2D contour plots of the interaction of relevant predictors were drawn.

## 3. Results and Discussion

### 3.1. Microstructure Analysis

**Figure 4:**
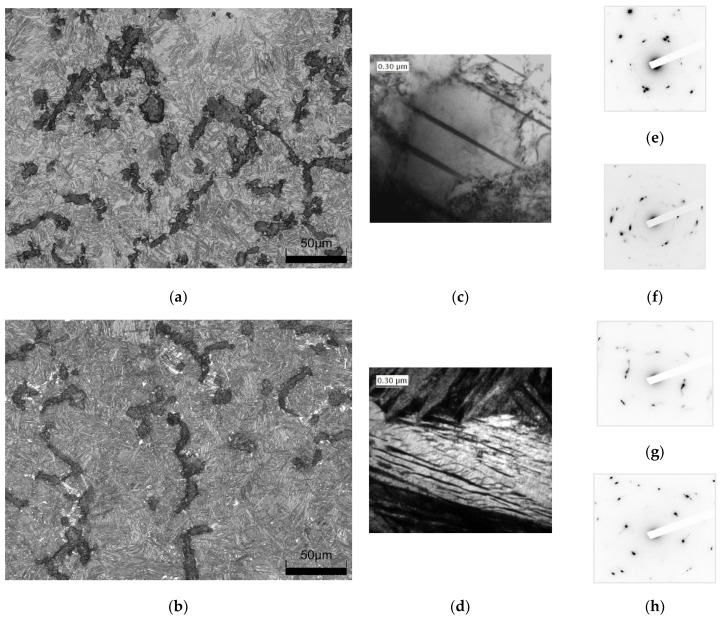
Microstructure of vermicular cast iron after heat treatment: (**a**) cast iron austenitized at 890 °C for 90 min and austempering at 390 °C for 90 min (Experiment No. 3 according to Table 1); (**b**) cast iron austenitized at 890 °C for 150 min and austempering at 290 °C for 150 min (Experiment No. 13). Structure obtained on TEM showing (**c**) austenite in the sample from Experiment No. 3; (**d**) a mixture of ferrite and austenite in the sample from Experiment No. 13. Electron diffraction photos of cast iron (Exp. No. 3): (**e**) austenite; (**f**) ferrite; and from Experiment No. 13: (**g**) austenite; (**h**) ferrite.

An Olympus DSX1000 digital microscope was used to describe the morphology of graphite precipitates in cast iron. To obtain additional information on the structure of AVGI, observations were made using a JEOL transmission electron microscope with an accelerating voltage of 300 kV. The results of these observations are presented in Figure 4. The microstructure obtained after heat treatment is ausferrite, which consists of acicular ferrite and carbon-saturated austenite. Two types of the resulting matrix are shown, differing in the shape of the ferrite needles and the amount of austenite present. In Figure 4a, the cast iron from Experiment Number 3 exhibits longer ferrite needles and a higher proportion of residual austenite compared to the sample from Experiment 13 (see Figure 4b). The formation, proportion, and shape of ferrite and austenite have been described in detail in the study [51]. The article [47] presents the morphology of graphite precipitates found in the iron in question and provides data on the area, perimeter, and number of graphite precipitates with respect to form factor classes, among others. In addition, the percentage of residual austenite in selected samples was determined using TEM (Table 3). Example images of cast iron microstructures after heat treatment are shown in Figure 4.

### 3.2. EDS Analysis

**Figure 5:**
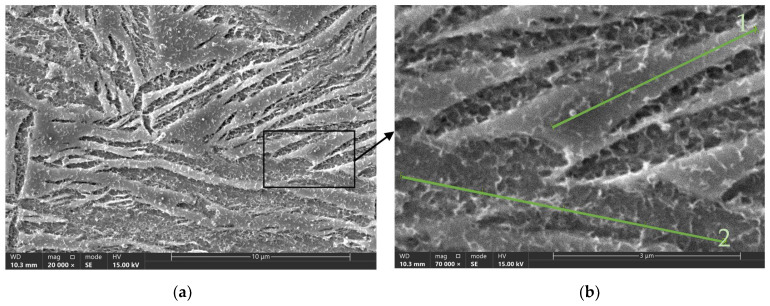
Scanning electron microscope images of samples from Experiment Number 4 (see Table 2). (**a**) Magnification 20,000×; (**b**) magnification 70,000× along with measurement green lines along which the chemical composition was determined.

The distribution of elements was examined using an Energy Dispersive Spectroscopy detector installed in an Axia ChemisSEM scanning electron microscope from ThermoFisher Scientific (Waltham, MA, USA). In particular, attention was paid to the distribution of elements in austenite and ferrite. For this, measurement lines were used along the precipitates of these components (see Figure 5), and the composition was examined over specific sections. The results are presented in Table 4. It should be noted that magnesium accumulated in austenite, while ferrite had a higher copper content.

### 3.3. Mechanical Properties of Cast Iron

**Figure 6:**
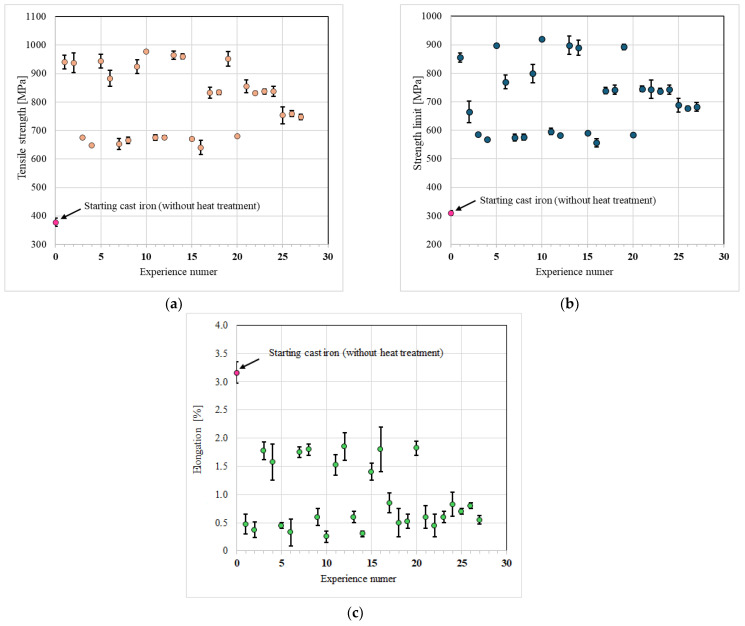
Mechanical properties of heat-treated vermicular cast iron classified according to the order of planned experiments; (**a**) tensile strength; (**b**) yield point; (**c**) elongation.

The results of measuring the properties of compacted graphite iron subjected to heat treatment are shown in Figure 6. By evaluating the mechanical properties of the cast iron before heat treatment and then comparing them with the mechanical properties of the cast iron following the heat treatment, it was possible to assess the effectiveness of the austempering operations carried out. The tensile strength of the baseline cast iron was about 340 MPa. After heat treatment, this value increased to 800 MPa on average. As for yield strength, the value for cast iron in the poured state was about 310 MPa, and after treatment, the average of all measurements increased to an average of about 700 MPa. The elongation of the cast iron samples in the pre-treatment state was 3.15% and about 0.9% after treatment.

Heat treatment, consisting of austempering, causes a change in the original structure of the baseline cast iron, which is reflected in the mechanical properties of the cast iron after heat treatment, among others. The highest tensile strength (ca. 978 MPa; Experiment 10) is found in cast iron austenitized at 960 °C for 90 min and then austempered at 290 °C for 150 min. The lowest R_m_ value (ca. 640 MPa; Experiment 16) is found in cast iron austenitized at 960 °C for 150 min and then austempered at 390 °C for 150 min. The relation of the yield strength of cast iron to the parameters of austempering is analogous to that of tensile strength. The largest R_0.2_ value of ca. 920 MPa was exhibited by the cast iron from Experiment 10, while the smallest (ca. 560 MPa) was from Experiment 16.

### 3.4. Impact of Heat Treatment Parameters on Rm

**Figure 7:**
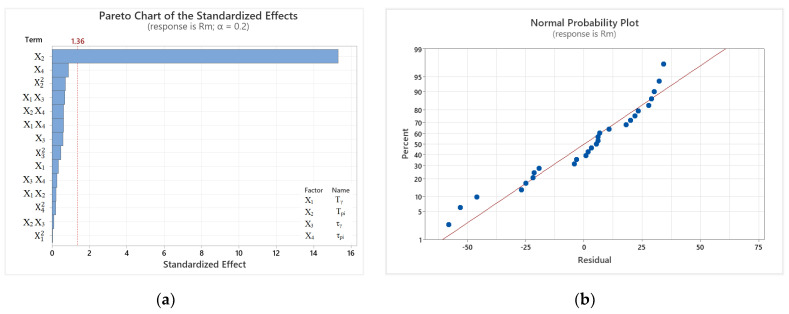
Graphs obtained for R_m_ at given heat treatment parameters: (**a**) Pareto chart of standardized effects; (**b**) normal probability plot of the residuals.

Figure 7a shows a Pareto chart of standardized effects obtained from analysis of variance (ANOVA) on main effects, primary effects interaction squared, and two-factor interaction. Only one interaction model was found to be significant at α = 0.2. According to the resulting model calculation, T_pi_ is the most relevant. Furthermore, the value of the coefficient of determination (R^2^) was relatively high (at 95.20%) and close to the value of the R^2^ adjusted (R^2^ adj) = 89.59%, which implies the linearity of the regression model. The suitability of the determined model for forecasting the size of R_m_ was evidenced by the high values of R^2^ predicted (R^2^-pred) obtained for the reliability of the selected model at 81.98%. The regression equation of the full model, describing the effect of cast iron heat treatment parameters on R_m_, takes the following form:

(2) $$R_{m}\;=1242-0.8\;X_{1}+3.55\;X_{2}+2.6\;X_{3}-5.2\;X_{4}+0.0007\;X_{1}^{2}-0.00688\;X_{2}^{2}+0.0126\;X_{3}^{2}+0.0053\;X_{4}^{2}-0.00118X_{1}X_{2}\;-0.00637\;X_{1}X_{3}+0.00565\;X_{1}X_{4}-0.00063\;X_{2}X_{3}-0.00404\;X_{2}X_{4}+0.0028\;X_{3}X_{4}$$

where in the formula *R_m_* is the calculated value of tensile strength [MPa]; *X*_1_—austenitization temperature (T_γ_) [°C]; *X*_2_—austempering temperature (T_pi_) [°C]; *X*_3_—austenitization time (τ_γ_) [min]; *X*_4_—austempering time (τ_pi_) [min].

From the Pareto chart, it can be observed that the austempering temperature is the most statistically significant factor. Among the insignificant factors, we can include the other three primary effects, namely austempering time, austenitizing time, and austenitizing temperature. It can be observed that T_γ_ has no significant impact on the mathematical model; however, the interaction with austenitizing time and with austempering time were the fourth and sixth variables presented in the Pareto chart. The strongest two-way interaction can be observed in the third position, and it relates to the austempering temperature squared. The least effective was the quadratic interaction of austenitizing temperature. Figure 7b shows a normal probability plot of the residuals, which approximately follows a straight line, indicating a normal distribution.

**Figure 8:**
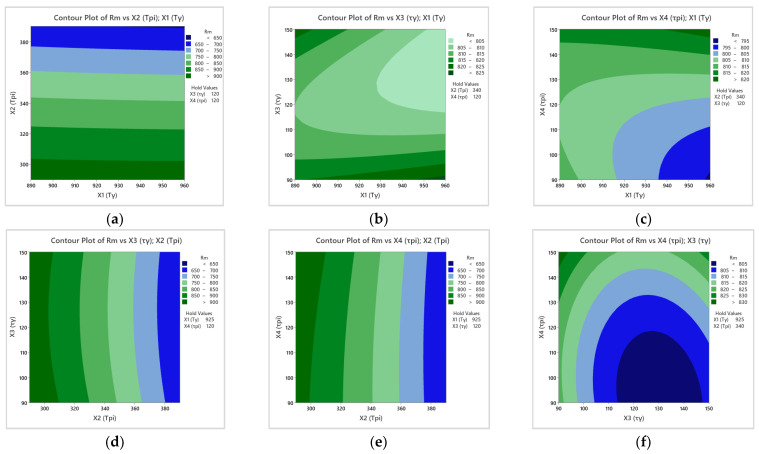
The influence of heat treatment parameters on Rm. The figure shows the relationship between the following heat treatment parameters: (**a**) T_γ_ and T_pi_; (**b**) T_γ_ and τ_γ_; (**c**) T_γ_ and τ_pi_; (**d**) T_pi_ and τ_γ_; (**e**) T_γ_ and τ_pi_; (**f**) τ_γ_ and τ_pi_.

Two-dimensional plots (see Figure 8) of the response surface show the impact of two heat treatment parameters on the tensile strength of cast iron. When analyzing the plots, special attention was paid to the parameter most relevant to the model in question, namely T_pi_. It can be seen from the curves in Figure 8a,d,e that as the austempering temperature increases, the material’s strength value increases. From Figure 8a, for example, one can see that at a T_pi_ between 380 and 400 °C, the tensile strength of cast iron will be around 650–700 MPa. There are no relations between the T_pi_ and other parameters. Increasing the austempering time results in decreased R_m_ (see Figure 8b,e,f).

### 3.5. Impact of Heat Treatment Parameters on R_0.2_

The equation of the full regression model describing the impact of cast iron heat treatment parameters on yield strength takes the following form:

(3) $$\begin{array}{c} R_{0.2}=1926+0.6\;X_{1}-2.9\;X_{2}+8.3\;X_{3}-15.0\;X_{4}-0.0028\;X_{1}^{2}-0.0024\;X_{2}^{2}+0.0001\;X_{3}^{2}-0.0049\;X_{4}^{2}+0.00504\;X_{1}X_{2}-\\ 0.0039\;X_{1}X_{3}+0.0236\;X_{1}X_{4}-0.01046\;X_{2}X_{3}-0.01237\;X_{2}X_{4}-0.0072\;X_{3}X_{4} \end{array}$$

where in the formula R_0.2_ is the calculated value of the yield strength [MPa]; *X*_1_—austenitization temperature (T_γ_) [°C]; *X*_2_—austempering temperature (T_pi_) [°C]; *X*_3_—austenitization time (τ_γ_) [min]; *X*_4_—austempering time (τ_pi_) [min].

**Figure 9:**
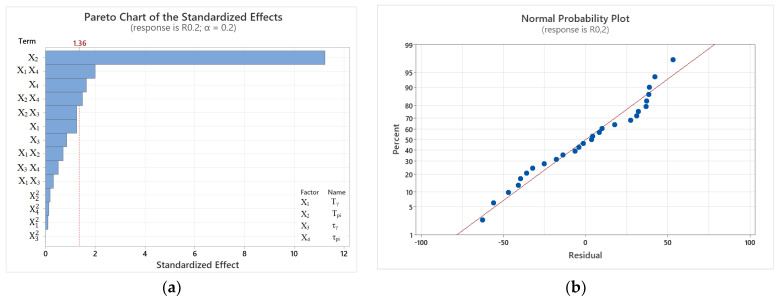
Graphs obtained for R_0,2_ at given heat treatment parameters: (**a**) Pareto chart of standardized effects; (**b**) normal probability plot of the residuals.

The value of the model’s coefficient of determination was 92.12%. The values of the adjusted and predicted determination coefficients were 82.93% and 48.95%, respectively. The determined model has a rather low coefficient of determination, which is due to the relatively large number of coefficients. The Pareto chart (Figure 9a) shows the absolute values of the impact factors. The vertical line indicates the critical value for the “t” test to assess the significance of the impact of the given factors on the output variable. It should be stressed that, at the assumed 80% confidence level, the impact of austempering temperature (T_pi_), the interaction of austenitizing temperature and austempering time (T_γ_ τ_pi_), austempering time (τ_pi_), and the interaction of austempering temperature and time (T_pi_τ_pi_) are significant. The coefficient determining the strength of the interaction of T_γ_, as well as the interaction of T_pi_ and τ_γ,_ are at a significance level exceeding the acceptable level by 0.03. The least significant primary factor was found to be austenitization time. The quadratic interactions of the input variables in question were the least effective. Virtually all points (residuals) on the normal probability plot of residuals (Figure 9b) lie very close to the line, which means that the residuals follow a normal distribution.

**Figure 10:**
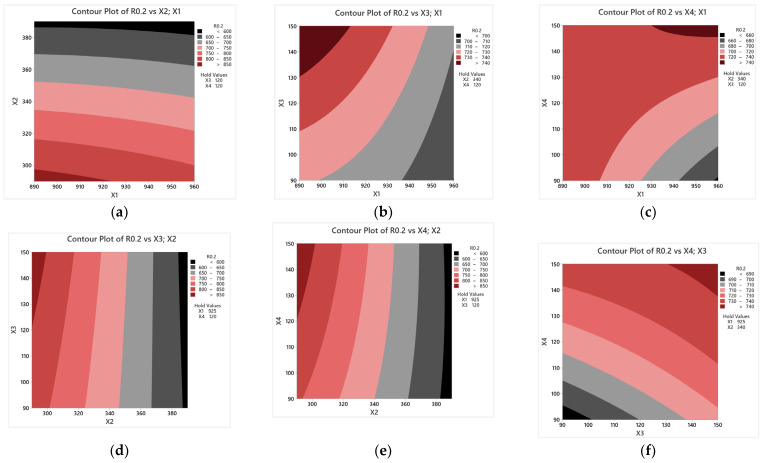
The influence of heat treatment parameters on the yield strength of cast iron. The figure shows the relationship between the following heat treatment parameters: (**a**) T_γ_ and T_pi_; (**b**) T_γ_ and τ_γ_; (**c**) T_γ_ and τ_pi_; (**d**) T_pi_ and τ_γ_; (**e**) T_γ_ and τ_pi_; (**f**) τ_γ_ and τ_pi_.

Figure 10 shows the impact of austenitizing as well as austempering temperature and time on R_0.2_ in the form of two-dimensional graphs. When analyzing the plots, special attention was paid to the most significant parameter. The austempering temperature, as in the case of R_m_, has a significant impact on the R_0.2_ of the cast iron, as evidenced by Figure 10a,d,f. As this temperature increases, the R_0.2_ increases as well. Figure 10b shows a very significant relation in the proposed mathematical model. In order to achieve high yield strength, the austenitizing temperature should be reduced, while the austenitizing time should be increased.

### 3.6. Impact of Heat Treatment Parameters on A_5_

The regression equation, taking into account the impact of all the heat treatment parameters discussed on the elongation of compacted graphite iron, takes the following form:

(4) $$\begin{array}{c} A_{5}=32.6-0.010\;X_{1}-0.1643\;X_{2}-0.0039\;X_{3}-0.0100\;X_{4}-0.000009\;X_{1}^{2}+0.000190\;X_{2}^{2}-0.000106\;X_{3}^{2}+0.000005\;X_{4}^{2}+\\ 0.000054\;X_{1}X_{2}+0.000032\;X_{1}X_{3}+0.000023\;X_{1}X_{4}+0.000006\;X_{2}X_{3}-0.000026\;X_{2}X_{4}-0.000025\;X_{3}X_{4} \end{array}$$

where in the formula *A*_5_ is the calculated elongation value [%]; *X*_1_—austenitization temperature (T_γ_) [°C]; *X*_2_—austempering temperature (T_pi_) [°C]; *X*_3_—austenitization time (τ_γ_) [min]; *X_4_*—austempering time (τ_pi_) [min].

The coefficient of multiple correlation, at 96.87% in this case, indicates a strong linear relationship between the dependent variable and the vector of independent variables. The adjusted coefficient is 93.21%. The corrected coefficient of determination indicates that 80.36% of the variation in results is consistent with the determined model.

**Figure 11:**
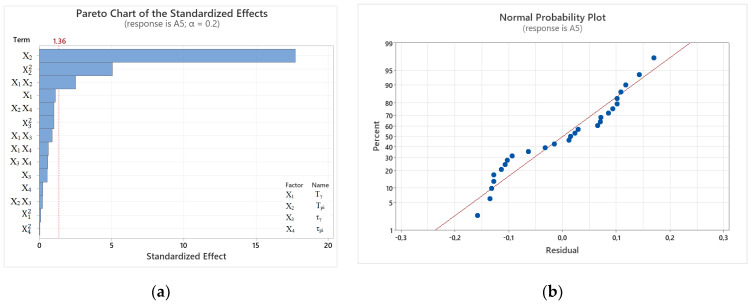
Charts obtained for the elongation dependence of cast iron for the adopted heat treatment parameters: (**a**) Pareto chart of standardized effects; (**b**) normal probability plot of the residuals.

After ANOVA analysis of the interaction of primary effects, squared, and the two-way interaction, a Pareto chart is shown for the resulting A_5_ of cast iron (Figure 11a). It shows the absolute values of the impact factors. The vertical line indicates the critical value for the *t*-test to assess the significance of the impact of the given factor on the output variable. At the assumed 80% confidence level (red line), only one of the main effects, namely **T_pi_**, is significant. The parameters that proved to be insignificant were T_γ_, τ_γ,_ and τ_pi_. Among the quadratic parameters, the significant parameter is the product of austempering temperatures, while the poorest, i.e., not significantly affecting the A_5_ of the alloy, was the austempering time squared. Among the parameters with two-way interaction, austenitizing temperature together with austempering temperature (T_γ_ T_pi_) obtained a *p*-Value of 0.026; the interaction of T_pi_ with τ_γ_ had the least impact. In Figure 11b, the points are arranged along a distribution function that is a straight line, indicating that the observed values follow a normal distribution.

**Figure 12:**
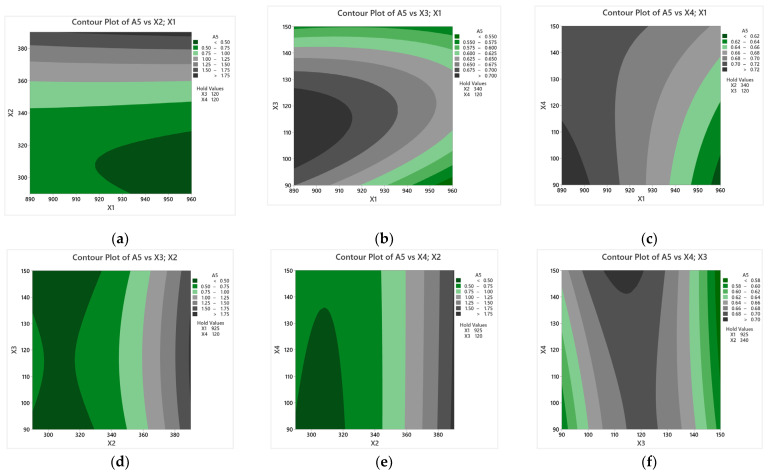
The influence of heat treatment parameters on the elongation of cast iron. The figure shows the relationship between the following heat treatment parameters: (**a**) Tγ and Tpi; (**b**) Tγ and τγ; (**c**) Tγ and τpi; (**d**) Tpi and τγ; (**e**) Tγ and τpi; (**f**) τγ and τpi.

Figure 12 shows the impact of heat treatment parameters on A_5_ in the form of two-dimensional graphs. As per the legend, the dark gray areas specify the parameters at which maximum elongation is achievable. A significant single-factor parameter in the present case is T_pi_, both squared and with a correlation with the austenitizing temperature. The relationship of the latter is illustrated in Figure 12a. Figure 12a,d,e show a significant increase in the elongation of cast iron when tempered at temperatures above 340 °C.

### 3.7. Response Optimization

**Figure 13:**
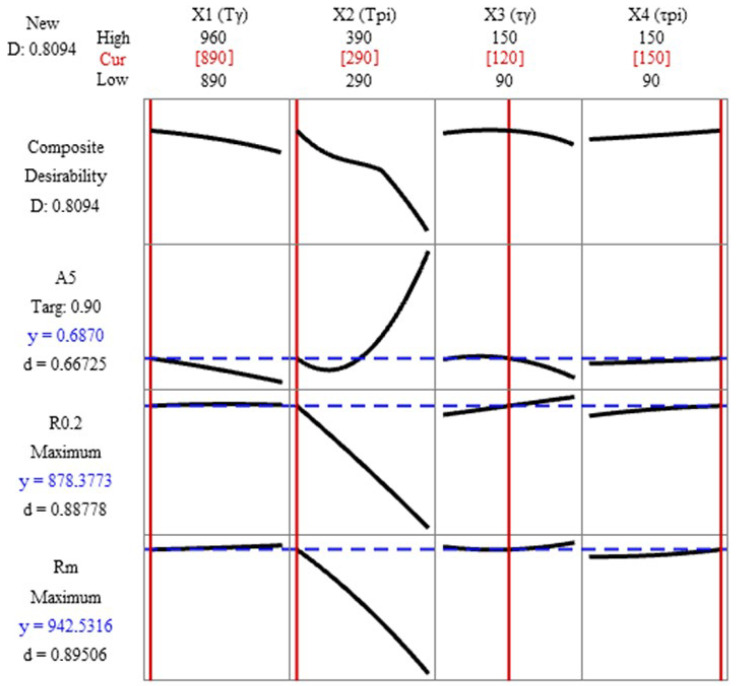
Desirability function applied in multiple responses (Minitab 21.4.3.0 Statistical Software).

For multi-criteria optimization of heat treatment parameters and their impact on the mechanical properties of cast iron, the RSM approach was used to identify the impact of each heat treatment parameter. Variable limits that achieve the maximum of each response are shown as “Cur.”. The goal of the optimization was to obtain the highest possible values for R_m_ and R_0.2_, assuming an A_5_ of the cast iron of about 0.7%. In the case of the material strength data, the determined desirability is at a satisfactory level of 80.94%. When analyzing the effect of the T_γ_, it can be seen that increasing the austenitizing temperature affects the deterioration of elongation, but at the same time, it is not significant for R_m_ and R_0.2_. Therefore, it can be concluded that this temperature can be reduced to an acceptable minimum, that is, about 850 °C. In the studied case, the optimal temperature was 890 °C. In the case of austempering temperature (T_pi_), increasing it causes a decrease in R_m_ and R_0.2_ with a simultaneous increase in elongation (A_5_). Analysis of the plot (see Figure 13) shows that the elongation in the T_pi_ ranges from 290 °C to approx. 310 °C decreases from approx. 0.69% to approx. 0.58%, and already above 310 °C it increases. As for the austenitizing time (τ_γ_), the optimal time is approx. 110 min. Increasing τ_γ_ results in a slight increase in R_0.2_, so the optimal setting was assumed to be closer to the middle of the range of the analyzed time. The austempering time (τ_pi_) shows minimal impact on the strength properties of compacted graphite iron. To summarize, in order to achieve the following properties for the alloy—R_m_ = 942 MPa, R_0.2_ = 878 MPa, and A_5_ = 0.7%, which would provide high values of desirability (d) of 0.89, 0.89, and 0.67, respectively—the heat treatment of cast iron should be carried out at the following parameters: T_γ_ = 890 °C; T_pi_ = 290 °C; τ_γ_ = 120 min; and τ_pi_ = 150 min.

**Figure 14:**
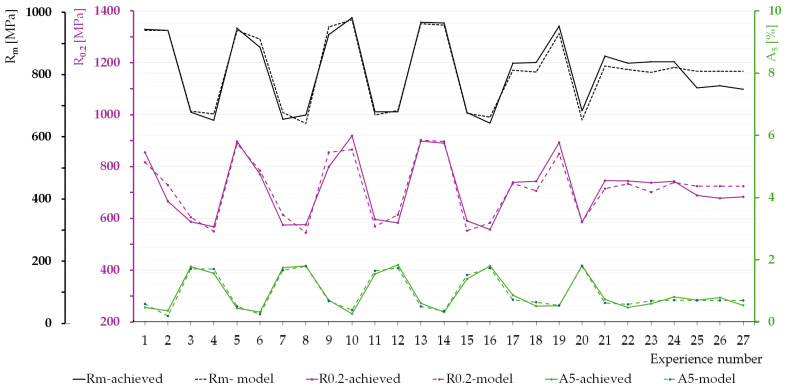
Comparison of the obtained strength results with the proposed mathematical models.

To verify the accuracy of the adopted regression models and the optimal heat treatment parameters determined based on them, Figure 14 presents the values of the considered mechanical properties of vermicular cast iron (R_m_, R_0.2_, and A_5_) obtained from the regression models compared with the corresponding data from experimental tests. It should be noted that the results show the best fit for elongation, followed by tensile strength, and the least accurate fit for yield strength.

In analytical research and development activities related to the production of vermicular cast iron, the provided information can help optimize the heat treatment process, reduce the number of tests, and shorten project timelines. It should be emphasized that experimental research methodology (Modern Experimental Design) and optimization are crucial for obtaining consistent results, improving production process efficiency, and minimizing costs. The experimental approach allows for a more precise determination of the relationships between process parameters and the mechanical properties of the material, which is essential for the further development of vermicular cast iron production technology.

## 4. Conclusions

Heat treatment of compacted graphite iron involving austempering in a manner analogous to that used in the manufacture of ADI alters the material structure, increasing tensile strength and yield strength while decreasing elongation. The microstructure after such treatment consists of acicular ferrite and residual austenite. Depending on the austempering temperature, the proportion of austenite changes. The lower this temperature is, the less austenite there is. As T_pi_ increases, the proportion of austenite increases.

Based on the analysis of variance and the analysis of multi-criteria optimization of heat treatment parameters of compacted graphite iron in relation to the obtained mechanical properties of compacted graphite iron, the following should be noted:

1. The proposed mathematical model for the impact of heat treatment parameters on the tensile strength of cast iron showed a predicted coefficient of determination of about 82% (with R^2^ adj = 90%). The R_m_ value has a significant impact on the proposed model due to one parameter, namely the austempering temperature;
2. The predicted coefficient of determination, with the model specifying R_0.2_, was about 50% (with R^2^ adj = 83%). The significant coefficients in this case are austempering temperature (T_pi_), the interaction of austenitizing temperature and austempering time (T_γ_ τ_pi_), austempering time (τ_pi_), and the interaction of austempering temperature and time (T_pi_τ_pi_) are significant;
3. In the case of polynomial regression determining the relation of cast iron elongation to heat treatment parameters, the predicted coefficient of determination was about 80% (with R^2^ adj = 93%). The significant parameters are the austempering temperature (T_pi_), the square of the quenching temperature (T_pi_ T_pi_), the two-way interaction of the austenitizing temperature together with the austempering temperature (T_γ_ T_pi_);
4. The goal of optimizing the impact of heat treatment parameters on the mechanical properties of compacted graphite iron was to achieve maximum tensile strength and maximum yield strength while maintaining elongation at about 0.7%. The assumed desirability of optimization was about 81%. When aiming to achieve a tensile strength of about 940 MPa, a yield strength of about 880 MPa, and an elongation of about 0.7%, compacted graphite iron should be heat-treated according to the following parameters Tγ = 890 °C; Tpi = 290 °C; τγ = 120 min.; and τpi = 150 min.

## Figures and Tables

**Table 1:** Chemical composition (wt.%) of vermicular cast iron [47].

C	Si	Mn	Cu	P	S	Mg
3.27	2.80	0.203	0.98	0.0515	0.0200	0.0166

**Table 2:** Factors of the designed experiment.

No	Output Factors				No	Output Factors			
	X_1_ (T_γ_)	X_2_ (T_pi_)	X_3_ (τ_γ_)	X_1_ (T_γ_)		X_1_ (T_γ_)	X_2_ (T_pi_)	X_3_ (τ_γ_)	X_1_ (T_γ_)
1	890	290	90	90	17	890	340	120	120
2	960	290	90	90	18	960	340	120	120
3	890	390	90	90	19	925	290	120	120
4	960	390	90	90	20	925	390	120	120
5	890	290	150	90	21	925	340	90	120
6	960	290	150	90	22	925	340	150	120
7	890	390	150	90	23	925	340	120	90
8	960	390	150	90	24	925	340	120	150
9	890	290	90	150	25	925	340	120	120
10	960	290	90	150	26	925	340	120	120
11	890	390	90	150	27	925	340	120	120
12	960	390	90	150					
13	890	290	150	150					
14	960	290	150	150					
15	890	390	150	150					
16	960	390	150	150					

**Table 3:** Results of measurements of the volume fraction of retained austenite in heat-treated vermicular cast iron.

No	Heat Treatment Parameters				Average Share of Retained Austenite [%]
	X_1_ (T_γ_) [°C]	X_2_ (T_pi_) [°C]	X_3_ (τ_γ_) [min]	X_4_ (τ_pi_) [min]	
4	960	390	90	90	37
8	960	390	150	90	37
11	890	390	90	150	26
15	890	390	150	150	26
17	890	340	120	120	23
18	960	340	120	120	33
19	925	290	120	120	15
22	925	340	150	120	30
23	925	340	120	90	21
25	925	340	120	120	28

No—defined in accordance with Table 2; X_1_ (T_γ_)—austenitization temperature; X_2_ (T_pi_) austempering temperature; X_3_ (τ_γ_)—austenitization time; X_4_ (τ_pi_)—austempering time.

**Table 4:** Results of linear EDS analysis along the straight lines shown in Figure 5b.

Element	Line 1					Line 2				
	Weight %									
	Line Point:									
	1	2	3	4	5	1	2	3	4	5
C	10.00	11.50	9.50	9.60	9.00	11.10	9.40	8.80	10.40	9.50
Mg	0.10	0.10	0	0.10	0.20	0	0	0	0	0
Si	2.60	2.50	2.80	2.70	2.70	2.60	2.50	2.60	2.50	2.40
Mn	0.20	0.40	0	0.70	0.70	0.20	0.20	0.00	0.60	0.40
Fe	86.90	83.70	86.80	85.50	85.70	84.70	86.50	87.00	85.20	85.70
Cu	0.30	1.80	0.90	1.30	1.60	1.30	1.40	1.70	1.30	1.90

## Data Availability

The original contributions presented in the study are included in the article, further inquiries can be directed to the corresponding author.
